# Evaluation of Multiple-Scale 3D Characterization for Coal Physical Structure with DCM Method and Synchrotron X-Ray CT

**DOI:** 10.1155/2015/414262

**Published:** 2015-03-10

**Authors:** Haipeng Wang, Yushuang Yang, Jianli Yang, Yihang Nie, Jing Jia, Yudan Wang

**Affiliations:** ^1^Institute of Theoretical Physics and Department of Physics, Shanxi University, Taiyuan, Shanxi 030006, China; ^2^CSIRO, Private Bag Box 33, Clayton, VIC 3169, Australia; ^3^State Key Laboratory of Coal Conversion, Institute of Coal Chemistry, Chinese Academy of Sciences, Taiyuan, Shanxi 030001, China; ^4^College of Physics & Electronics Engineering, Shanxi University, Taiyuan, Shanxi 030006, China; ^5^Shanghai Institute of Applied Physics, Chinese Academy of Sciences, Shanghai 201800, China

## Abstract

Multiscale nondestructive characterization of coal microscopic physical structure can provide important information for coal conversion and coal-bed methane extraction. In this study, the physical structure of a coal sample was investigated by synchrotron-based multiple-energy X-ray CT at three beam energies and two different spatial resolutions. A data-constrained modeling (DCM) approach was used to quantitatively characterize the multiscale compositional distributions at the two resolutions. The volume fractions of each voxel for four different composition groups were obtained at the two resolutions. Between the two resolutions, the difference for DCM computed volume fractions of coal matrix and pores is less than 0.3%, and the difference for mineral composition groups is less than 0.17%. This demonstrates that the DCM approach can account for compositions beyond the X-ray CT imaging resolution with adequate accuracy. By using DCM, it is possible to characterize a relatively large coal sample at a relatively low spatial resolution with minimal loss of the effect due to subpixel fine length scale structures.

## 1. Introduction

Physically, a coal sample can be grouped in three compositions: coal matrix (organic composition), minerals (inorganic compositions), and pores. The physical structure of coal is relevant to the sorption and diffusion of coal-bed methane (CBM) in coal-bed as well as the transformation of minerals in coal processing [1–7]. The distributions of coal compositions (physical structures) are inherently heterogeneous and multiscale, ranging from nanometer to millimeter scale and above. Multiple-scale three-dimensional characterization of coal physical structure is helpful for clean and high efficient utilization of coal. It is also useful for obtaining fundamental data to establish three-dimensional (3D) fluid transportation model in coal matrix during enhanced coal-bed methane (ECBM) process.

X-ray CT imaging can nondestructively obtain 3D materials microstructure information. Numerous works related to the application of X-ray CT in coal microstructure characterization have been done previously. Verhelst et al. [8] and Simons et al. [9] investigated the correlation between the tomodensity with the real physical bulk density of coal compositions. Van Geet et al. [10, 11] demonstrated the application of microfocus CT and the use of dual energy approach. Karacan and Okandan [12, 13], Mazumder et al. [14], and Yao et al. [15, 16] also studied the distribution of different compositions in coal sample by X-ray CT imaging. At present, microfocus CT combined with dual-energy method and image segmentation has been an important method for quantitative characterization of the physical structure of coal.

Although remarkable progresses have been made by previous researchers, there are still some limitations with this technique in coal characterization. Firstly, in most cases the characterization of the coal physical structure is a multiscale problem. For example, pore sizes ranging from several hundred micrometers to several nanometers all contribute to coal-bed methane transportation. However, there exists a scale limitation with existing 3D characterization techniques such as X-ray CT: it is not possible for the sample size to go beyond 10^4^ times of the scanned voxel size. That is to say, for a sample with a size of centimeters, the imaging resolution size could not be smaller than a micrometer. For a factor 10 increase in resolution, the CT data size is increased by 10^3^ times. In addition to this, the X-ray exposure increases as the 4th power with resolution or 10^4^ times with a factor 10 increase in resolution. The dataset size, exposure induced sample temperature increase, and image acquisition time would become unpractical for a moderate further improvement in resolution. In order to obtain multiple-scale information of various materials microstructure, micro-CT was usually combined with other high resolution techniques during the characterization process, such as the combination of micro-CT and nanofocus CT [17], scanning electron microscope (SEM) [18], and focused ion beam (FIB) [19]. Despite the successes, the high resolution techniques can only provide details for a small region of a sample or a small sample, while for some nonhomogenous materials a large sample would be more accurate representation statically. Secondly, the fine length scale structures in the coal sample produce effective mixing of multiple compositions at the X-ray CT voxels. This leads to a nonunique relationship between material compositions and X-ray CT image grey scale. This makes it inadequate to use the conventional image segmentation technique to resolve the compositions.

Recently, a data-constrained modeling (DCM) approach [20, 21] has been developed. It combines the multispectrum X-ray CT data with a statistical mechanical model to resolve the coexistence of multiple compositions in the same voxel (the partial volume effect). It has been successfully used to characterize microstructures of different materials.

Wang et al. [22] have applied this approach to characterize an anthracite coal sample collected from Yangquan coal mine, China. The mineral compositions, coal matrix, and pores were divided into four groups based on their X-ray absorption characteristics. The volume fractions of each composition group in individual CT voxel were obtained using the DCM approach. Fine compositional structures which are smaller than the CT imaging resolution contribute to voxel partial volumes. Through using DCM, the compositions smaller than the X-ray CT spatial resolution can be characterized quantitatively. This method has opened new opportunity for multiple-scale 3D characterization of coal.

Despite the success of previous work, the sensitivity of the results on imaging spatial resolution is still unknown. The purpose of this study is to establish the spatial resolution sensitivity for coal microstructure characterization using DCM and multiple X-ray CT datasets acquired at different spatial resolution. It forms the basis for multiple-scale characterization of coal samples.

## 2. Experimental

### 2.1. Sample and CT Experiment

The coal sample used in this study is the same one as used by previous study and the mineral compositions, coal matrix, and pores were divided into four groups (as listed in Table 1) based on their X-ray absorption characteristics [22]. That is, those compositions were treated as one group when their X-ray absorption coefficients as functions of X-ray energy were approximately linearly dependent on each other.

**Figure 1:**
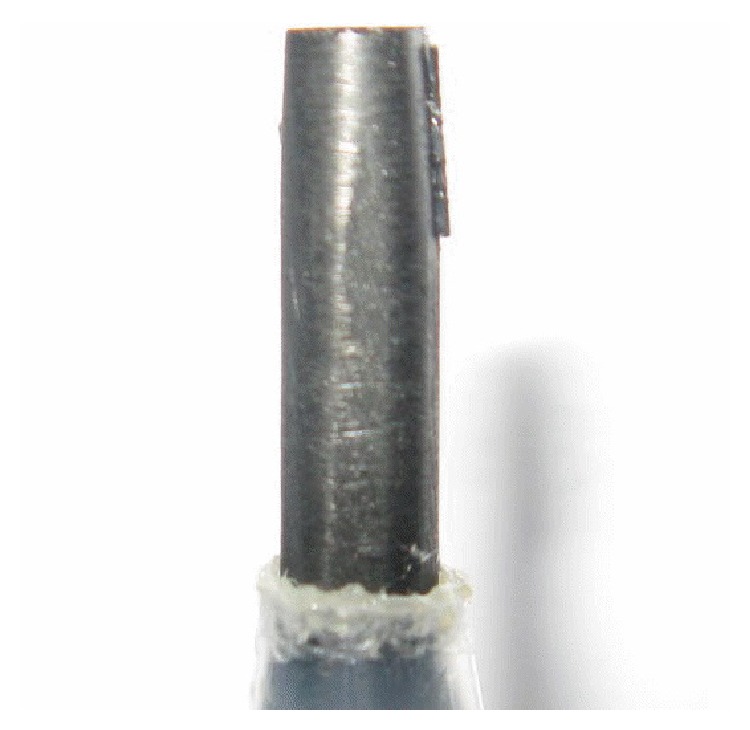
Coal sample used in this study.

For convenience of image alignment with different resolutions, a short nylon wire was stuck to the sample (Figure 1) as a marker. There was no obvious pore on the sample surface.

The X-ray CT projection data was acquired on the BL13W beam-line at the Shanghai Synchrotron Radiation Facility (SSRF). A Si (111) double-crystal monochromator was used, which produces a quasi-monochromatic X-ray beam with a relative bandwidth smaller than 5 × 10^−3^. The excellent monochromaticity of synchrotron X-ray makes the correlation between the tomodensity with the physical density of the coal sample more easy to be established. An Optique Peter X-ray CCD detector with a native pixel size of 7.4 × 7.4 *μ*m^2^ was used in the experiment. With a 2× optical lens in front of the CCD, the sample was imaged with an imaging resolution of 3.7 *μ*m. Monochromatic X-ray beam energies of 14 kev, 18 kev, and 22 kev were selected. A total of 900 projection images were collected at each X-ray beam energy. The angular spacing was 0.2° between each two projections for a total rotation angle of 180°. After this, the sample was imaged again with an imaging resolution of 11.84 *μ*m using a 1.25× optical lens and 2 × 2 pixels binning. The position of sample and the orientation of the sample axis were not changed when the optical lens was changed. This can ensure the same portion of the sample was imaged at the abovementioned two imaging resolutions. For imaging resolution of 11.84 *μ*m, the same X-ray beam energies were used as for imaging resolution of 3.7 *μ*m. A total of 360 projection images were collected at each X-ray beam energy. The projection images were acquired with an angular spacing 0.5° for a total sample rotation angle of 180°. The dark-current and flat-field images were also acquired at the beginning and at the end of each scan which are used to normalize the projection images before CT reconstruction. For imaging resolution size of 3.7 *μ*m, a total of 1395 image slices with the size of 1947 × 1947 pixels were reconstructed using the X-TRACT software [23] at each beam-energy. For image resolution size of 11.84 *μ*m, a total of 697 image slices with the size of 973 × 973 pixels were reconstructed at each beam energy.

### 2.2. DCM Approach

The sample was represented by a cubic grid of *N* = *l* × *l* × *n* voxels in the DCM model, where *l* × *l* is the total pixel size of a CT slice and *n* is the number of selected slices. The voxel size in the DCM model is identical to the voxel size of reconstructed X-ray CT slices. The DCM model is to minimize the following objective function at each voxel [20, 22]:

(1) $$\begin{array}{c} \Gamma_{}=\overset{3}{\underset{q=1}{\sum }}{\left[\overset{3}{\underset{m=0}{\sum }}\mu_{}^{\left(q,m\right)}v_{}^{\left(m\right)}-{\hat{\mu }}_{}^{\left(q\right)}\right]}_{}^{2}+\overset{3}{\underset{m=0}{\sum }}v_{}^{\left(m\right)}\varepsilon_{}^{\left(m\right)}. \end{array}$$

In (1), the *q*  ( = 1,2, 3) corresponds to the experimental CT data at beam energies 14, 18, and 22 keVs, respectively; *m*  ( = 0,1, 2,3) represent the compositional groups A, B, C, and D, respectively; *v* ^(*m*)^ is volume fractions of composition group *m* on the voxel; *μ* ^(*q*,*m*)^ is the linear absorption coefficients of composition group *m* at the X-ray energy *q*; *ε* ^(*m*)^ is the phenomenological chemical potentials of composition group *m*; ${\hat{\mu }}_{}^{\left(q\right)}\;\;(q=1,2,3)$ is X-ray CT measured linear absorption coefficient values on the voxel. The following constraints should be applied in minimization of (1):

(2)  0≤v(0),v(1),v(2),v(3)≤1 v0+v1+v2+v3=1.

For the DCM approach, the volume fractions of each of the composition groups can be obtained by solving (1) and (2) using a constrained search algorithm.

For the purpose of comparing the DCM reconstructed compositions at different resolutions, a subset of 96 slices with pixel size of 3.7 *μ*m and a subset of 30 slices with pixel size of 11.84 *μ*m were selected at each X-ray beam energy. The selected slices with different voxel sizes correspond to the same physical portion of the coal sample. The subset was selected with minimal image defects. The composition distributions at the two resolutions were computed with the DCM approach. For quantitative comparisons of DCM reconstructed compositions in different regions, seven cubical subregions of the selected slices were used. The size of the subregions is so selected such that each of them contains an integer number of voxels at both resolutions. This ensures that the subregions at different resolutions are corresponding to the same portion of the coal sample. The size of a subregion is 96 × 96 × 96 pixels at a resolution 3.7 *μ*m and 30 × 30 × 30 pixels at a resolution 11.84 *μ*m, respectively. During the DCM computation process, the pore self-energy was selected as 0.0000805 to reproduce the previous measured porosity of the coal sample, and other parameters were the same as previous study [22].

## 3. Results and Discussion

**Figure 2:**
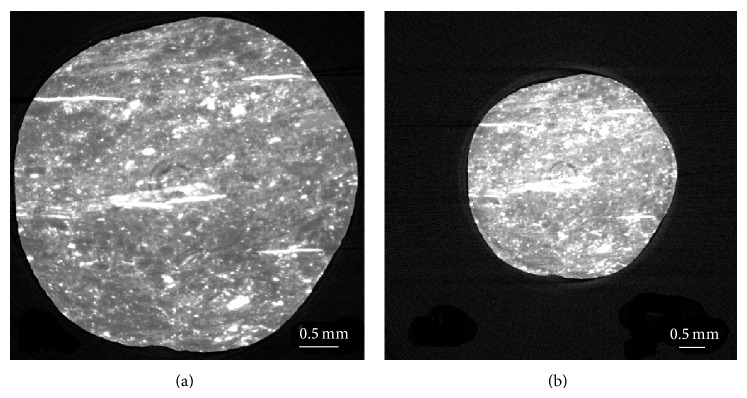
The original reconstructed CT slices at different spatial resolutions: (a) at resolution of 3.7 *μ*m; (b) at resolution of 11.84 *μ*m.

Figure 2 shows two typical reconstructed CT slices at spatial resolution of 3.7 *μ*m and 11.84 *μ*m respectively. They correspond to the same sample slice. A voxel in Figure 2(b) is equivalent to 3.2^3^ voxels in Figure 2(a). The X-ray linear absorption coefficient of a voxel in Figure 2(b) is the average of those 3.2^3^ voxels in Figure 2(a). There are obvious ring artifacts existing in the images, which are difficult to be eliminated completely during the CT reconstruction, although a ring-defect filter had been applied. In these two images, the grey areas are mainly coal matrix; the white areas indicate high X-ray absorption, which represent minerals. From Figure 2, it is easy to see that although the two slices ((a) and (b)) look similar, the details are clearer in the image with the higher resolution. The reason for this is that Figure 2(a) contained more pixels than Figure 2(b), which enable a more detailed characterization for the sample.

Table 2 is the DCM computed average composition volume fractions at the two CT imaging resolutions. In Table 2, Area 0 represents DCM computed results of the whole selected subset slices of the coal sample. Areas 1–7 represent DCM computed results of the seven subregions.

It can be seen from Table 2 that the computed volume fractions of different composition groups for the two image resolutions are very close. The difference of the computed volume fractions for coal matrix and pore is less than 0.3%. For the mineral compositions group, the difference is less than 0.17%. It should be note that though the maximal difference for pore in the eight areas is 0.28%, the average difference is only 0.14%. The minor differences may be related to the experimental noise during the projection image acquisition process and the ring artifact in the CT slices. The difference of coal matrix is higher than other compositions, since the volume fraction of coal matrix in coal is far more than other compositions. The results indicate that the DCM approach can be used to characterize coal microstructures using low resolution CT data with minimal loss of fine subvoxel structure effects. For the same physical volume in the coal sample, about 30 times more computational time is required for the image resolution at 3.7 *μ*m than that at 11.84 *μ*m as shown in Table 2.

**Figure 3:**
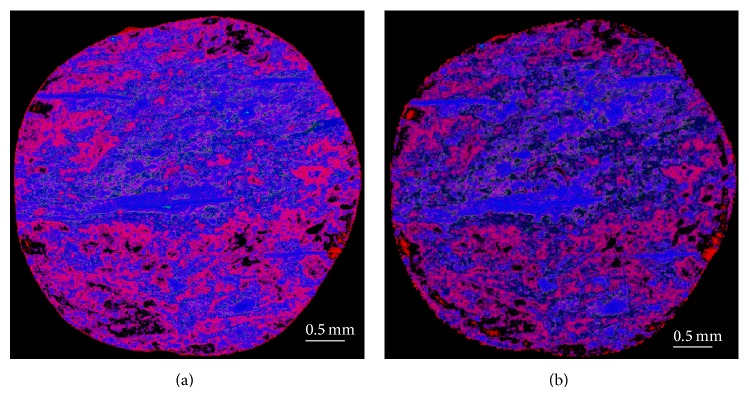
DCM reconstructed compositional distributions: (a) at resolution of 3.7 *μ*m; (b) at resolution of 11.84 *μ*m. Pores are displayed as red; group C minerals are displayed as blue; group D minerals are displayed as green. The pixel display intensity for each colour is proportional to the appropriate compositional volume fraction. Coexistence of multiple compositions in a voxel is shown as colour mixing. The coal matrix is not displayed.

Figures 3(a) and 3(b) show the DCM computed compositional distributions at two different image resolutions. They are on the same portion of the sample as in Figures 2(a) and 2(b). Although Figures 3(a) and 3(b) are quite similar overall, the composition of group D (green area) is more visible in Figure 3(a) than that in Figure 3(b). The reason for this is that the display intensity of fine particles at a coarse display resolution is suppressed as their small volume fractions tend to be overwhelmed by other compositions. In other words, the small regions of group D appear as diluted and spread out in Figure 3(b).

**Figure 4:**
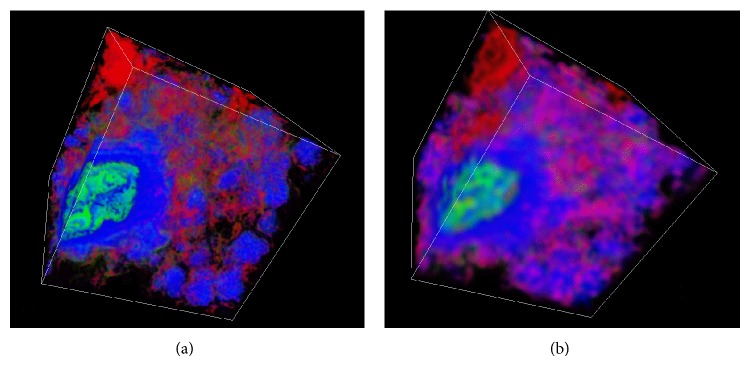
3D physical structure of Area 1 at different spatial resolutions: (a) at resolution of 3.7 *μ*m; (b) at resolution of 11.84 *μ*m. The same colouring scheme is used as in Figure 3. The displayed sample size is 355.2 × 355.2 × 355.2 *μ*m^3^.

Figures 4(a) and 4(b) are the 3D physical structures of the coal sample corresponding to Area 1 at different spatial resolutions. It can be seen from these two images that the pores are distributed as a 3D connected network, while group C mineral composition is distribution as clusters. The apparent intensity of red color (pore) in Figure 4(a) is higher than that in Figure 4(b), for the same reason as has been mentioned in Figure 3. The visual effect of such dispersed compositions can be enhanced by multiplying their volume fractions values by a suitable factor (>1).

Although the numerical results indicate that DCM can model the partial volume effect reliably, it should be noted that it does not increase the spatial resolution. That is, it does not give any additional information about how the multiple compositions are spatially distributed inside a voxel. Investigations on the impact of subvoxel spatial distributions still need high resolution techniques. Besides this, the DCM still cannot to be used for lab-CT. The X-ray of lab-CT is polychromatic, which make the correlation between the tomodensity with the physical density of sample more difficult to be established.

## 4. Conclusions

The 3D physical structure of a Yangquan coal sample is obtained using the DCM nonlinear optimization approach with multienergy synchrotron X-ray CT at different image resolutions. The distributions of pores, coal matrix, and minerals in the same region of the coal sample have been quantitatively reconstructed at different spatial resolutions. By comparing average volume fractions of mineral phases and pores in these subregions in the sample, it is concluded that the DCM computed results were consistent under the two resolutions. For materials with structures span over multiple length scales such as coal, it has been difficult to characterize them using the existing 3D X-ray CT technique due to limited spatial dynamical range of the technique. If one wish to resolve the fine length scale structures, the sample size has to be reduced accordingly that would reduce its statistical representativeness. The results presented in this paper indicate that using a large sample would not necessarily lose subvoxel effect by using the DCM approach, although, with higher image resolution, spatial distributions of compositions whose size is smaller than the pixel size can be resolved more precisely. With a reduced spatial resolution, the computational efficiency is improved.

## Figures and Tables

**Table 1:** Compositions groups of coal sample.

Groups	A	B	C	D
Including compositions	Pore	Coal matrix	Illite, quartz, and kaolinite	Chlorite and titania

**Table 2:** DCM computed average composition group volume fractions.

Area	Imaging resolution (*μ*m)	Computed volume fractions (%)				Computed time (s)
		Group A	Group B	Group C	Group D	
Area 0	3.7	4.48	87.80	7.58	0.14	9.06 × 10^5^
	11.84	4.37	88.00	7.51	0.12	6.55 × 10^4^

Area 1	3.7	3.30	86.90	9.40	0.40	2447
	11.84	3.39	86.90	9.31	0.40	70

Area 2	3.7	5.25	89.30	5.40	0.05	2428
	11.84	4.97	89.60	5.36	0.07	79

Area 3	3.7	3.27	85.40	11.10	0.23	2274
	11.84	3.36	85.40	11.00	0.24	71

Area 4	3.7	4.82	89.10	6.02	0.06	2366
	11.84	4.63	89.40	5.85	0.12	72

Area 5	3.7	4.14	88.50	7.22	0.14	2547
	11.84	4.25	88.20	7.37	0.18	70

Area 6	3.7	4.35	88.20	7.28	0.17	2415
	11.84	4.21	88.40	7.16	0.23	73

Area 7	3.7	4.52	89.20	6.17	0.11	2386
	11.84	4.41	89.40	6.07	0.12	70

## References

[1] Alexeev A. D., Feldman E. P., Vasilenko T. A. (2010). Kinetics of methane desorption from coal nano-and mesostructures. *Energy and Fuels*.

[2] Pillalamarry M., Harpalani S., Liu S. (2011). Gas diffusion behavior of coal and its impact on production from coalbed methane reservoirs. *International Journal of Coal Geology*.

[3] Han F. S., Busch A., Krooss B. M., Liu Z. Y., Yang J. L. (2013). CH_4_ and CO_2_ sorption isotherms and kinetics for different size fractions of two coals. *Fuel*.

[4] Everson R. C., Neomagus H. W. J. P., Kaitano R. (2011). The random pore model with intraparticle diffusion for the description of combustion of char particles derived from mineral- and inertinite rich coal. *Fuel*.

[5] Hol S., Spiers C. J., Peach C. J. (2012). Microfracturing of coal due to interaction with CO_2_ under unconfined conditions. *Fuel*.

[6] Yuan W. N., Pan Z. J., Li X. (2014). Experimental study and modelling of methane adsorption and diffusion in shale. *Fuel*.

[7] Golab A., Ward C. R., Permana A., Lennox P., Botha P. (2013). High-resolution three-dimensional imaging of coal using microfocus X-ray computed tomography, with special reference to modes of mineral occurrence. *International Journal of Coal Geology*.

[8] Verhelst F., David P., Fermont W., Jegers L., Vervoort A. (1996). Correlation of 3D-computerized tomographic scans and 2D-colour image analysis of Westphalian coal by means of multivariate statistics. *International Journal of Coal Geology*.

[9] Simons F. J., Verhelst F., Swennen R. (1997). Quantitative characterization of coal by means of microfocal X-ray computed microtomography (CMT) and color image analysis (CIA). *International Journal of Coal Geology*.

[10] Van Geet M., Swennen R., Wevers M. (2000). Quantitative analysis of reservoir rocks by microfocus X-ray computerised tomography. *Sedimentary Geology*.

[11] Van Geet M., Swennen R., David P. (2001). Quantitative coal characterisation by means of microfocus X-ray computer tomography, colour image analysis and back-scattered scanning electron microscopy. *International Journal of Coal Geology*.

[12] Karacan C. Ö., Okandan E. (2000). Fracture/cleat analysis of coals from Zonguldak Basin (northwestern Turkey) relative to the potential of coalbed methane production. *International Journal of Coal Geology*.

[13] Karacan C. O., Okandan E. (2001). Adsorption and gas transport in coal microstructure: investigation and evaluation by quantitative X-ray CT imaging. *Fuel*.

[14] Mazumder S., Wolf K.-H. A. A., Elewaut K., Ephraim R. (2006). Application of X-ray computed tomography for analyzing cleat spacing and cleat aperture in coal samples. *International Journal of Coal Geology*.

[15] Yao Y. B., Liu D. M., Che Y., Tang D. Z., Tang S. S., Huang W. H. (2009). Non-destructive characterization of coal samples from China using microfocus X-ray computed tomography. *International Journal of Coal Geology*.

[16] Yao Y. B., Liu D. M., Cai Y. D., Li J. Q. (2010). Advanced characterization of pores and fractures in coals by nuclear magnetic resonance and X-ray computed tomography. *Science China Earth Sciences*.

[17] Patterson B. M., Henderson K. C., Gibbs P. J., Imhoff S. D., Clarke A. J. (2014). Laboratory micro- and nanoscale X-ray tomographic investigation of Al–7at.%Cu solidification structures. *Materials Characterization*.

[18] Quey R., Suhonen H., Laurencin J., Cloetens P., Bleuet P. (2013). Direct comparison between X-ray nanotomography and scanning electron microscopy for the microstructure characterization of a solid oxide fuel cell anode. *Materials Characterization*.

[19] Yazzie K. E., Williams J. J., Phillips N. C., de Carlo F., Chawla N. (2012). Multiscale microstructural characterization of Sn-rich alloys by three dimensional (3D) X-ray synchrotron tomography and focused ion beam (FIB) tomography. *Materials Characterization*.

[20] Yang Y. S. (2012). A data-constrained non-linear optimisation approach to compositional microstructure prediction. *Lecture Notes in Information Technology*.

[21] Yang S., Tulloh A., Chen F., Chu C. Dcmlite Software. https://data.csiro.au/dap/landingpage?pid=csiro:9448.

[22] Wang H. P., Yang Y. S., Wang Y. D., Yang J. L., Jia J., Nie Y. H. (2013). Data-constrained modelling of an anthracite coal physical structure with multi-spectrum synchrotron X-ray CT. *Fuel*.

[23] Gureyv T., Nesterets Y., Thompson D., Wilkins S., Stevenson A., Sakellariou A., Oleg C. Toolbox for advanced X-ray image processing.

